# CLPTM1L modulates membrane lipid rafts to promote tumor EGFR signaling

**DOI:** 10.1093/lifemeta/loag012

**Published:** 2026-05-20

**Authors:** Dejian Pang, Xuan Yang, Xinyao Li, Zixuan Xue, Xincan Hou, Kemu Xiao, Yun Yang, Guanlin Wang, Tong-Jin Zhao, Junfeng Bi

**Affiliations:** Shanghai Key Laboratory of Metabolic Remodeling and Health, Institute of Metabolism and Integrative Biology, Fudan University, Shanghai 200438, China; Shanghai Key Laboratory of Metabolic Remodeling and Health, Institute of Metabolism and Integrative Biology, Fudan University, Shanghai 200438, China; Shanghai Key Laboratory of Metabolic Remodeling and Health, Institute of Metabolism and Integrative Biology, Fudan University, Shanghai 200438, China; Shanghai Key Laboratory of Metabolic Remodeling and Health, Institute of Metabolism and Integrative Biology, Fudan University, Shanghai 200438, China; Shanghai Key Laboratory of Metabolic Remodeling and Health, Institute of Metabolism and Integrative Biology, Fudan University, Shanghai 200438, China; Shanghai Key Laboratory of Metabolic Remodeling and Health, Institute of Metabolism and Integrative Biology, Fudan University, Shanghai 200438, China; Shanghai Key Laboratory of Metabolic Remodeling and Health, Institute of Metabolism and Integrative Biology, Fudan University, Shanghai 200438, China; Shanghai Key Laboratory of Metabolic Remodeling and Health, Institute of Metabolism and Integrative Biology, Fudan University, Shanghai 200438, China; Shanghai Key Laboratory of Metabolic Remodeling and Health, Institute of Metabolism and Integrative Biology, Fudan University, Shanghai 200438, China; State Key Laboratory of Metabolic Dysregulation & Prevention and Treatment of Esophageal Cancer, Innovation Center of Basic Research for Metabolic-Associated Fatty Liver Disease, Ministry of Education of China, Tianjian Laboratory of Advanced Biomedical Sciences, Academy of Medical Sciences, Zhengzhou University, Zhengzhou, Henan 450001, China; Shanghai Key Laboratory of Metabolic Remodeling and Health, Institute of Metabolism and Integrative Biology, Fudan University, Shanghai 200438, China

**Keywords:** membrane lipid remodeling, lipid rafts, CLPTM1L, GPI-anchored proteins, RTK signaling, glioblastoma

## Abstract

The plasma membrane dynamically organizes into specialized lipid domains to sustain cell proliferative signaling, yet the regu­latory mechanisms driving this process, especially during tumor progression, remain poorly understood. Here, we uncover cleft lip and palate transmembrane protein 1-like protein (CLPTM1L), an endoplasmic reticulum-localized lipid scramblase, as a critical regulator of membrane raft formation and the epidermal growth factor receptor (EGFR)-mediated proliferative signaling in cancer. High *CLPTM1L* expression was significantly associated with poor patient survival in glioblastoma (GBM), the most aggressive brain cancer. Depletion of *CLPTM1L* disrupts cellular lipid homeostasis and results in a substantial loss of membrane raft components, including glycosphingolipids and glycosylphosphatidylinositol (GPI)-anchored proteins. The cell-surface level of EGFR, which colocalizes with raft marker GM1, is markedly reduced upon *CLPTM1L* loss. We show that CLPTM1L-mediated raft remodeling promotes EGFR signaling and drives cell proliferation in both cancer and non-cancer cells. In GBM mouse models, *CLPTM1L* depletion inhibits EGFR signaling and profoundly impairs orthotopic tumor growth. Our work establishes CLPTM1L as a key regulator of membrane raft domain formation and highlights its critical role in cancer proliferative signaling.

## Introduction

The plasma membrane of cells is a dynamic and heterogeneous bilayer structure composed primarily of lipids and proteins [1]. These components with unique biophysical properties can form ordered lipid domains, also named lipid rafts or micro/nanodomains, that serve as critical functional platforms for various cellular processes [2–4]. One example is that membrane rafts can facilitate clustering of growth factor receptors and other signal molecules on the cell membrane to process signal transduction for cell proliferation [5, 6]. The organization of raft domains can be regulated by lipid−lipid and lipid−protein interactions among specific sphingolipids, cholesterol, phospholipids, and raft proteins [2, 7, 8]. Despite their importance, how cells coordinately modulate membrane lipid metabolism and raft protein maturation to establish and maintain these functional domains, particularly in specific cellular processes, remains largely unexplored.

Sustained proliferative signaling is a hallmark of cancer [9], which drives cancer cells to undergo uncontrolled cell division and persistent proliferation. The plasma membrane is a key cellular structure for initiating these receptor-mediated signals [6]. Aberrant activation of growth factor receptors, particularly receptor tyrosine kinases (RTKs), has been identified as one of the major oncogenic drivers that induce proliferative signaling and progression in various cancers [9, 10]. Glioblastoma (GBM) is the most aggressive and lethal primary brain cancer in adults, with the epidermal growth factor receptor (EGFR) signaling characterized as the major driver of its progression. Recent evidence suggests lipid rafts as a signaling platform and hub for cancer cells [11]. RTKs, such as EGFR, interact with lipid rafts and assemble into clusters to trigger downstream signaling cascades [12, 13]. However, how specific alterations of membrane raft domains sustain tumor proliferative signaling remains elusive.

Dysregulated lipid metabolism plays critical roles in cancer progression [12]. Membrane raft-enriched lipids, including glycosphingolipids, cholesterol, and specific phospholipids, are involved in the raft formation, integrity, and function [2, 4, 7]. Cholesterol depletion, for instance, disrupts raft organization, leading to impaired RTK signaling in cancer cells [14, 15]. Our earlier work demonstrated that phospholipid saturation remodeling activates EGFR within the lipid rafts, promoting GBM growth [16–18]. Beyond these lipid components, glycosylphosphatidylinositol-anchored proteins (GPI-APs) represent an evolutionarily conserved family of raft-associated proteins, with unique lipid tails integrating into the outer leaflets of the cell membrane [19, 20]. GPI-APs can form nanoclusters with raft lipids and signaling molecules, facilitating critical cellular functions [8, 21, 22]. Tumor cells need to orchestrate the biosynthesis of functional membrane systems from limited nutrient resources to sustain their persistent proliferation [12, 23]. Yet, the metabolic remodeling of these membrane raft components and their functions in tumor proliferative signaling remain largely unclear.

In this study, we investigated the regulatory mechanisms underlying membrane raft lipid and protein remodeling in GBM cell lines and mouse models. We identified that cleft lip and palate transmembrane protein 1-like protein (CLPTM1L), an endoplasmic reticulum (ER)-localized scramblase, coordinately modulated cellular lipid homeostasis and membrane raft formation. This CLPTM1L-mediated regulation promoted EGFR signaling and promoted tumor progression.

## Results

### ER lipid scramblase CLPTM1L is highly expressed in cancer

**Figure 1:**
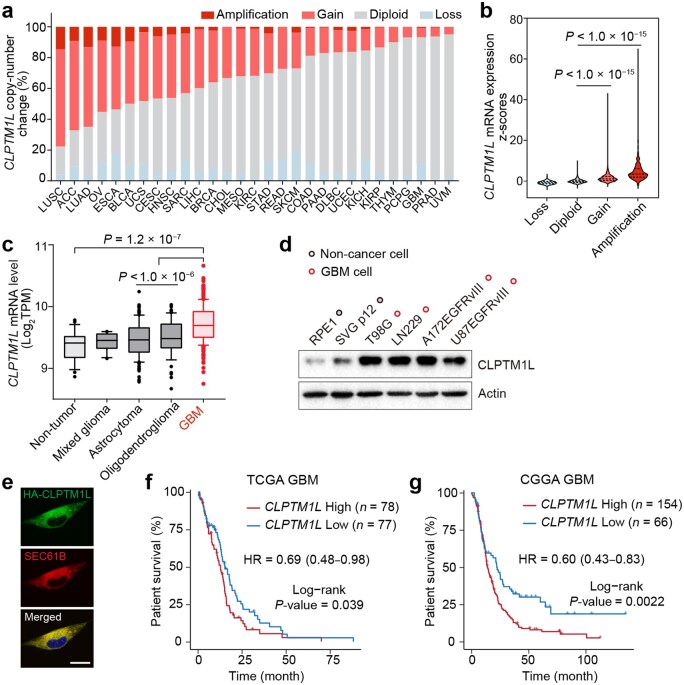
ER lipid scramblase CLPTM1L is highly expressed in cancer. (a) Frequency of *CLPTM1L* copy-number alterations in TCGA cancer types. (b) *CLPTM1L* mRNA level in tumor samples of patients with *CLPTM1L* copy-number variants. (c) *CLPTM1L* mRNA levels (log_2_TPM) in non-tumor tissues, LGG, and GBM. (d) CLPTM1L protein in non-cancer lines and cancer lines. (e) Colocalization of HA-tagged CLPTM1L and ER marker SEC61B-mScarlet in T98G cells. DAPI staining indicates cell nuclei. Scale bar: 20 µm. (f) Overall survival of patients with high or low *CLPTM1L* expression in TCGA GBM cohort. (g) Overall survival of patients with high or low *CLPTM1L* expression in the CGGA GBM + cohort. ANOVA followed by multiple comparison test in (b and c). Log-rank test in (f and g).

Scramblases and flippases regulate lipid translocation between membrane bilayers to maintain membrane homeostasis [24]. To identify regulators of membrane lipid remodeling during cell proliferation, we systematically analyzed the expression levels of all reported lipid scramblases and flippases [24, 25], in cancer samples and normal tissues using the Gene Expression Profiling Interactive Analysis (GEPIA) dataset [26]. We found that CLPTM1L, a newly identified lipid scramblase [25], showed significantly higher mRNA levels across multiple cancer types compared with normal tissues (Supplementary Fig. S1a). Notably, analysis of The Cancer Genome Atlas (TCGA) datasets revealed that the copy number of *CLPTM1L* was frequently gained or amplified in many cancer types, including lung cancer (lung squamous cell carcinoma [LUSC] and lung adenocarcinoma [LUAD]), adenoid cystic carcinoma (ACC), esophageal carcinoma, and GBM (Fig. 1a). This copy-number increase was significantly associated with elevated *CLPTM1L* mRNA at the pan-cancer level (Fig. 1b), suggesting that genomic alterations may drive its high expression. We next analyzed *CLPTM1*L expression in GBM, the most aggressive brain tumor driven by RTK signaling of the plasma membrane. We observed significant upregulation of *CLPTM1L* in GBM compared with non-tumor tissues (*P *= 1.7 × 10^−7^) or low-grade glioma (LGG) (*P *< 1.0 × 10^−6^) (Fig. 1c; Supplementary Fig. S1b). Further validation by western blot and quantitative reverse-transcription poly­merase chain reaction (qRT-PCR) analyses confirmed that both *CLPTM1L* mRNA and protein were overexpressed in GBM cancer cell lines, but not in the nontumor cells (Fig. 1d; Supplementary Fig. S1c).

To verify that CLPTM1L is indeed an ER-localized protein, we performed staining for its subcellular localization in GBM cells, demonstrating colocalization of HA-tagged CLPTM1L with ER marker SEC61B (Fig. 1e). Given the high expression of *CLPTM1L* in GBM cells, we next evaluated its clinical relevance by analyzing the association between *CLPTM1L* mRNA expression and overall survival in patients across two independent GBM datasets. Patients with high *CLPTM1L* mRNA levels exhibited significantly shorter survival compared to those with low levels in TCGA GBM dataset (*P *= 0.039) and the Chinese Glioma Genome Atlas (CGGA) GBM dataset (*P *= 0.0022) (Fig. 1f and g). In further detailed analyses of GBM subcohorts, no significant association was observed between *CLPTM1L* expression and overall survival in patients with *TP53* mutations (Supplementary Fig. S1d). These results together demonstrate that ER-localized lipid scramblase CLPTM1L is highly expressed in GBM and correlates with poor patient survival, highlighting its potential functional importance in cancer.

### CLPTM1L promotes the proliferation of cancer cells and nontumor cells

**Figure 2:**
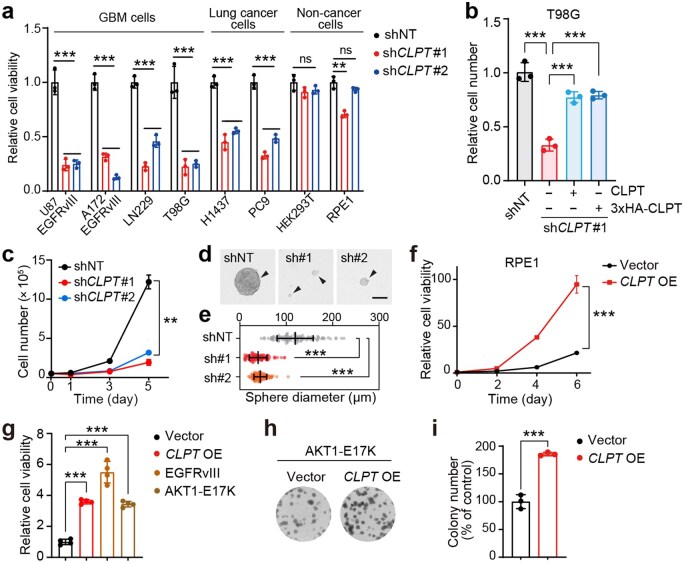
CLPTM1L promotes cell proliferation in cancer and non-cancer cells. (a) Relative cell viability of non-cancer and cancer cell lines. (b) Relative cell number of T98G cells with *CLPTM1L* shRNA knockdown and coding sequence rescue. (c) Relative cell proliferation curves of T98G cells with non-targeting or *CLPTM1L* shRNAs. (d) Representative GBM sphere images. Scale bar: 100 µm. (e) Quantification of sphere size in (d). (f) Cell viability curves of non-cancer RPE1 cells with vector or *CLPTM1L* overexpression. (g) Relative cell viability of RPE1 cells with *CLPTM1L* and indicated oncogene overexpression. (h) Colony formation of RPE1 cells. AKT1-E17K is a constitutively activated AKT1 mutation. (i) Quantification of colony numbers in (h). Data represent mean ± SD. ANOVA followed by Tukey’s multiple comparisons test in (a–c and e–g). Two-tailed Student’s *t*-test in (i). ns, not significant; **P < 0.01; ***P < 0.001.

To determine the role of CLPTM1L in cell proliferation, we performed short hairpin RNA (shRNA)-mediated knockdown in a panel of cancer and non-cancer cell lines (Supplementary Fig. S2a). *CLPTM1L* depletion remarkably suppressed the viability in four GBM cell lines but had minimal inhibitions in HEK293T and retinal pigment epithelium-1 (RPE1), two non-cancer cell lines (Fig. 2a; Supplementary Fig. S2b–c). GBM cells exhibited more severe viability inhibition than lung cancer cells to *CLPTM1L* depletion, suggesting a high dependency for GBM. Knockdown of *CLPTM1L* using UTR-targeting shRNAs dramatically inhibited T98G cell viability (Fig. 2b), which was significantly rescued by re-expressing its coding sequence (Fig. 2b). Further, we observed that *CLPTM1L* knockdown also blocked T98G cell proliferation (Fig. 2c) and severely impaired GBM sphere formation and growth (Fig. 2d and e). Conversely, overexpression of *CLPTM1L* significantly enhanced the colony formation in three GBM cell lines (Supplementary  Fig. S2d and e). To assess the proliferative-promoting role of CLPTM1L in non-cancer cells, we overexpressed *CLPTM1L* in the RPE1 cells, a human retinal pigment epithelial cell line, observing a striking increase in cell viability and cell numbers (Fig. 2f; Supplementary Fig. S2f). This effect was comparable to two well-characterized oncogenic mutants, EGFRvIII or AKT1-E17K (Fig. 2g). Notably, *CLPTM1L* overexpression nearly doubled colony formation in AKT1-E17K-expressing RPE1 cells (Fig. 2h and i), indicating a functional interaction between oncogenic mutants and CLPTM1L. Together, these results strongly suggest that CLPTM1L is a driver of cancer cell proliferation.

### CLPTM1L regulates lipid homeostasis and membrane raft lipid remodeling

**Figure 3:**
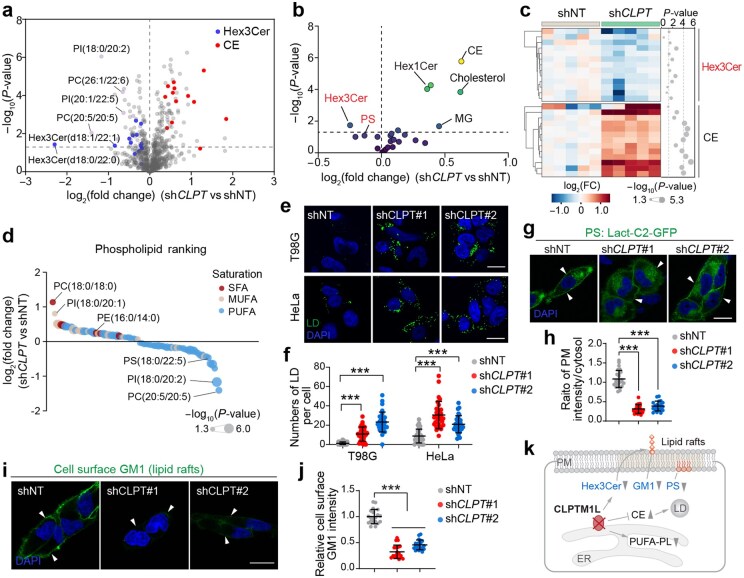
CLPTM1L regulates lipid homeostasis and promotes membrane raft lipid remodeling. (a) Volcano plots of fold changes of lipid abundance with *P*-values. (b) Enrichment analysis of significantly altered lipid species. (c) Heatmap of Hex3Cer and CEs. (d) Ranking plots of significantly changed phospholipids. (e and f) Lipid droplet staining and quantification. Scale bar: 20 µm. (g) Imaging of PS sensor, Lact-C2-GFP, in T98G cells. Scale bar: 20 µm. (h) Ratio of cell surface to total intensity of Lac-C2-GFP. (i and j) Cell surface staining of lipid raft marker GM1 by CTxB-488 conjugates in T98G cells. GM1 is a marker for lipid rafts, and DAPI staining indicates cell nuclei. (k) Schematic diagram of lipid changes in GBM cells upon *CLPTM1L* depletion. Data represent mean ± SD. Two-tailed Student’s *t*-test in (a–d). ANOVA followed by Tukey’s multiple comparisons test in (f, h, and j). ***P < 0.001.

CLPTM1L, as a lipid scramblase on the ER, exhibits scramblase activity to various phospholipid species [25], which could regu­late the membrane lipid asymmetry. To investigate the role of CLPTM1L in membrane lipid remodeling, we performed lipidomic analysis in T98G cells following *CLPTM1L* knockdown. Compared with the nontargeting control, *CLPTM1L* depletion induced significant alterations in whole-cell lipid profiles, particularly in sphingolipids, storage neutral lipids, and phospholipids (Fig. 3a and b; Supplementary Table S1). Specifically, Hex3Cer, a class of glycosphingolipids enriched in cell membrane and lipid raft domains, showed the most pronounced decrease among all lipid classes in *CLPTM1L*-depleted cells (Fig. 3a–c). Conversely, Hex1Cer, a precursor of Hex3Cer biosynthesis, was significantly increased (Fig. 3b), indicating a potential defect of Hex3Cer biosynthesis upon *CLPTM1L* depletion. Cholesterol and cholesterol esters (CEs) were also significantly elevated (Fig. 3b and c), while phosphatidylserine (PS) species were slightly reduced (Fig. 3b). Furthermore, analysis of significantly altered phospholipids indicated that di-saturated and monounsaturated fatty acids (MUFA)-containing species, such as phosphatidylcholine (PC) (18:0/18:0) and phosphatidylethanolamine (PE) (16:0/14:0), were significantly increased (Fig. 3d), whereas polyunsaturated fatty acids (PUFA)-containing phospholipid species, including PC (20:5/20:5), were significantly reduced in *CLPTM1L-*depleted cells (Fig. 3a and d; Supplementary Fig. S3a). Consistent with the observed increase in CEs, *CLPTM1L* knockdown significantly elevated lipid droplet numbers in both T98G and HeLa cells (Fig. 3e and f), further suggesting the function of CLPTM1L in maintaining cellular lipid homeostasis.

To examine the membrane raft lipid remodeling, we next employed fluorescent-labeled lipid probes for confocal imaging analysis. PS is an important anionic phospholipid enriched in the inner leaflet of the cell membrane and lipid rafts [27]. We investigated PS distribution by using PS sensor Lact-C2-GFP, showing that *CLPTM1L* depletion significantly reduced the ratio of plasma membrane signals relative to cytosol (Fig. 3g and h), indicating CLPTM1L-mediated regulation of PS membrane distribution. Given the observed decrease of membrane raft-enriched lipids, such as Hex3Cer and PS, we then tested whether *CLPTM1L* depletion could modulate membrane raft domain enrichment. Monosialotetrahexosylganglioside (GM1) is a subspecies of glycosphingolipids that reside in the outer leaflet of the plasma membrane and is used as a lipid marker of lipid rafts [28]. We next examined the membrane rafts in T98G cells by cell surface GM1 staining using CTxB-488 conjugates, and observed a dramatic reduction in cell surface GM1 signal in *CLPTM1L*-depleted T98G cells (Fig. 3i and j), indicating substantial membrane raft domain disruption upon CLPTM1L loss. These results together suggest that CLPTM1L plays a critical role in maintaining cellular lipid homeostasis and membrane raft domain formation (Fig. 3k).

### CLPTM1L modulates plasma membrane GPI-APs and EGFR

**Figure 4:**
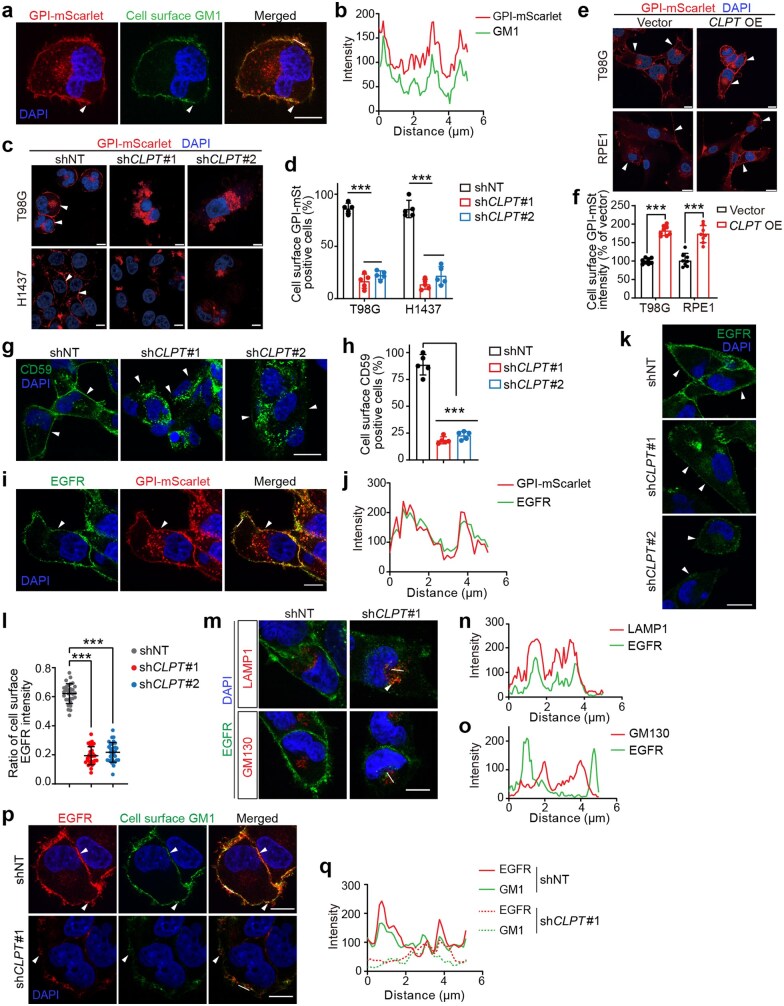
CLPTM1L modulates plasma membrane GPI-APs and EGFR. (a and b) Colocalization of lipid raft marker GM1 with GPI-mScarlet in T98G cells. Scale bar: 10 µm. (c and d) GPI-mScarlet in cancer cells with *CLPTM1L* knockdown. Scale bar: 5 µm. (e and f) GPI-mScarlet in T98G and RPE1 cells with *CLPTM1L* overexpression. Scale bar: 5 µm. (g and h) CD59 staining in T98G cells with *CLPTM1L* shRNA knockdown. Scale bar: 20 µm. (i and j) Colocalization of EGFR with GPI-mScarlet in T98G cells. Scale bar: 10 µm. (k and l) Total EGFR staining and cell surface EGFR quantification (ratio of cell membrane/total intensity) in T98G cells with *CLPTM1L* knockdown. Scale bar: 20 µm. (m) Staining of total EGFR and LAMP1 or GM130 in T98G cells. Scale bar: 10 µm. (n and o) Intensity quantification of ER and LAMP1 (n) or GM130 (o) along indicated lines in (m). (p) Staining of total EGFR and lipid raft marker GM1 in T98G cells. Scale bar: 10 µm. (q) Intensity quantification of EGFR and lipid raft marker GM1 along indicated lines in (p). Scale bar: 20 µm. DAPI staining indicates cell nuclei. Data represent mean ± SD. ANOVA followed by Tukey’s multiple comparisons test. ***P < 0.001.

Having revealed the role of CLPTM1L in regulating membrane lipids and raft domains, we next determined its impact on membrane raft proteins and oncogenic growth factor receptors in *CLPTM1L*-depleted cells. GPI-APs represent key membrane raft proteins [7], and CLPTM1L can also translocate GPI precursors on the ER to facilitate the maturation of GPI-APs, besides its scramblase activity to lipids [25]. To determine the involvement of CLPTM1L in GPI-AP biosynthesis and membrane localization in cancer cells, we designed a fluorescently labeled marker, GPI-mScarlet (Supplementary Fig. S3b). We observed significant colocalization of GPI-mScarlet with the lipid raft marker GM1 in T98G cells (Fig. 4a and b). Depletion of *CLPTM1L*, by two individual shRNAs, attenuated GPI-mScarlet signals on the plasma membrane of two cancer cell lines, which were significantly restored by expressing its coding sequence (Fig. 4c and d; Supplementary Fig. S3c and d). Accordingly, GPI-mScarlet signals showed marked accumulation in the ER in *CLPTM1L* knockdown cells (Supplementary Fig. S3e), indicating impaired transport to the plasma membrane. Further, depletion of PGAP2 or PGAP3, two key enzymes for modification of GPI-APs in the Golgi, similarly reduced the cell surface level of GPI-mScarlet (Supplementary Fig. S3f and g). Conversely, *CLPTM1L* overexpression increased the level of GPI-mScarlet on the plasma membrane in T98G and RPE1 cells (Fig. 4e and f). We next examined endogenous GPI-APs on the plasma membrane. Using a CD59-specific antibody, we found that *CLPTM1L* knockdown markedly reduced endogenous CD59 levels on the plasma membrane (Fig. 4g and h), whereas overexpression increased cell surface CD59 levels in T98G cells (Supplementary Fig. S3h), further supporting the role of CLPTM1L in promoting the membrane localization of GPI-APs.

We next tested whether CLPTM1L could regulate oncogenic RTKs within lipid rafts of GBM cells. EGFR is the major oncogenic driver of GBM, and its activation relies on the localization of membrane raft domains in GBM [16, 29]. Indeed, we observed significant colocalization between EGFR and GPI-mScarlet on the cell surface of T98G cells (Fig. 4i and j). We next assessed EGFR levels on the cell membrane following *CLPTM1L* depletion. We observed a significant reduction in EGFR levels on the plasma membrane of *CLPTM1L*-depleted cells (Fig. 4k and l). In these *CLPTM1L*-depleted cells, cytosolic EGFR puncta were observed to colocalize with the lysosomal marker lysosomal-associated membrane protein 1 (LAMP1), but not with Golgi markers GM130 or TGN46 (Fig. 4m–o; Supplementary Fig. S4a and b). Consistently, the treatment of chloroquine, a lysosomal inhibitor, resulted in a partial rescue of EGFR level in *CLPTM1L-*depleted cells (Supplementary Fig. S4c), suggesting a lysosome-dependent degradation of EGFR after *CLPTM1L* depletion. Further, EGFR showed significant colocalization with lipid raft marker GM1 in T98G cells (Fig. 4p and q), confirming its raft colocalization. *CLPTM1L* depletion led to marked reductions in both GM1 and EGFR levels on the plasma membrane of T98G cells (Fig. 4p and q), and puncta of EGFR negative with GM1 were observed on the cell membrane, suggesting CLPTM1L-mediated regulation of oncogenic EGFR. Collectively, these results indicate that CLPTM1L may play a critical role in maintaining the membrane raft abundance of EGFR in cancer cells.

### CLPTM1L promotes oncogenic EGFR signaling in GBM

**Figure 5:**
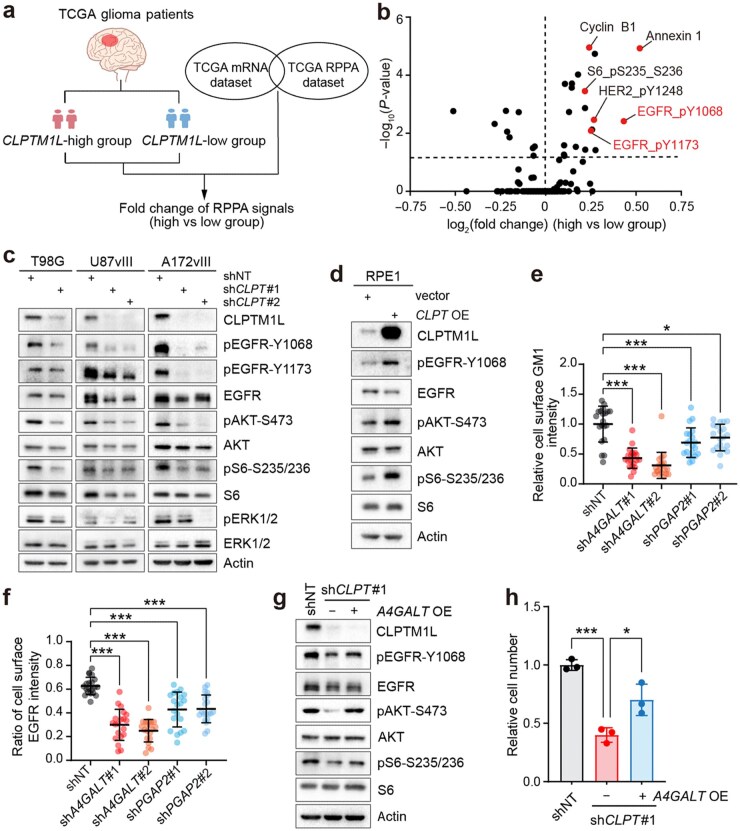
CLPTM1L promotes EGFR signaling in GBM. (a) Schematic diagram of RPPA signal analysis in TCGA glioma patients. Patients were categorized into two groups based on high or low *CLPTM1L* mRNA levels. (b) Volcano plots of fold change of RPPA signals (log_2_) in TCGA glioma patients with high *CLPTM1L* expression. (c) Western blot analysis of EGFR signaling in three GBM cell lines with *CLPTM1L* knockdown. (d) Western blot analysis of EGFR signaling in RPE1 cells with *CLPTM1L* overexpression. (e and f) Quantification of cell surface intensity of GM1 (e) and EGFR (f) in GBM cells with *A4GALT* or *PGAP2* knockdown. (g) Western blot analysis of EGFR signaling in *CLPTM1L*-depleted cells with or without *A4GALT* overexpression. (h) Relative cell number of A172vIII cells. Data represent mean ± SD. Two-tailed Student’s *t*-test in (b). ANOVA followed by Tukey’s multiple comparisons test in (e, f, and h). *P < 0.05; ***P < 0.001.

Membrane raft domains are essential for the activation of oncogenic RTK signaling in GBM [16, 17]. To unbiasedly profile the downstream pathways of CLPTM1L, we systematically analyzed the mRNA expression (RNA-seq) dataset and reverse phase protein arrays (RPPA) dataset of TCGA glioma patient cohorts (Fig. 5a). The patient cohort was stratified into two groups based on *CLPTM1L* expression, and the fold changes of signaling molecules between these two groups were calculated and plotted, accompanied by their corresponding *P*-values (Fig. 5b; Supplementary Table S2). Strikingly, elevated levels of multiple RTK signaling pathway markers, including pEGFR-Y1068, pEGFR-Y1173, pHER2-Y1248, and pS6-S235/236, were observed in *CLPTM1L*-high patients (Fig. 5b). Meanwhile, we found that cell cycle-related protein Cyclin B1 and Annexin 1, a membrane-localized phospholipid-binding protein, were also upregulated in patients with *CLPTM1L*-high expression (Fig. 5b). These results from patient datasets suggest that CLPTM1L may promote the oncogenic RTK signaling in GBM.

To further confirm this, we performed the shRNA knockdown in three GBM cell lines with endogenous wild-type EGFR or ectopic EGFRvIII expression. Consistently, genetic depletion of *CLPTM1L* dramatically suppressed the EGFR activity and downstream mechanistic target of rapamycin complex 1/2 (mTORC1/2) and extracellular signal-regulated kinase (ERK) signaling, as indicated by reduced pEGFR-Y1068, pEGFR-Y1173, pAKT-S473, pERK1/2, and pS6-S235/236 (Fig. 5c; Supplementary Fig. S4d). Conversely, overexpression of *CLPTM1L* enhanced the EGFR-mTORC1/2 signaling in non-cancer RPE1 cells (Fig. 5d), consistent with its role in promoting cell proliferation (Fig. 2f–i). This indicates that CLPTM1L is sufficient to drive EGFR-mediated cell proliferation in non-cancer cells.

To determine the role of membrane rafts in CLPTM1L-mediated proliferative signaling, we performed shRNA-mediated knockdown of alpha 1,4-galactosyltransferase (*A4GALT*) and post-GPI attachment to proteins 2 (*PGAP2*), which encode key enzymes for Hex3Cer biosynthesis (Supplementary Fig. S4e) and GPI-AP modi­fication (Supplementary Fig. S3b), respectively. Consistent with *CLPTM1L* depletion, the cell surface levels of EGFR and GM1 were significantly reduced in *A4GALT*- or *PGAP2*-depleted cells, with a more pronounced reduction observed upon *A4GALT* knockdown (Fig. 5e and f; Supplementary Fig. S4f). This result suggests that both Hex3Cer biosynthesis and GPI-AP maturation may contri­bute to the CLPTM1L-mediated signaling. Importantly, overexpression of *A4GALT* significantly restored both EGFR signaling and the viability of *CLPTM1L*-depleted cells (Fig. 5g and h). Collectively, these results indicate that CLPTM1L-mediated membrane raft remodeling promotes oncogenic EGFR signaling and GBM cell proliferation.

### *CLPTM1L* depletion suppresses EGFR signaling and tumor growth in mice

**Figure 6:**
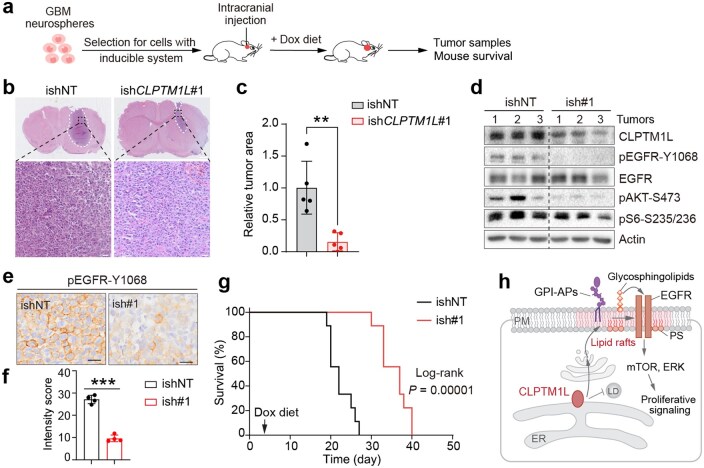
CLPTM1L depletion suppresses EGFR signaling and impairs orthotopic tumor growth. (a) Schematic overview of the orthotopic GBM tumor experiment using a doxycycline-inducible knockdown system. (b) H&E staining of brain sections. Scale bar for upper panels: 500 µm. Scale bar for lower panels: 20 µm. (c) Relative tumor area. (d) EGFR-mTOR signaling in tumor samples. (e and f) IHC staining of pEGFR-Y1068 in tumor sections. Scale bar: 20 µm. (g) Kaplan–Meier survival analysis of mice bearing GBM tumors with doxycycline diet administration. (h) Proposed model for CLPTM1L function. The ER lipid scramblase CLPTM1L modulates cellular lipid homeostasis and elevates plasma membrane glycosphingolipids and PS. This regulation of membrane raft glycosphingolipids coordinates with the membrane trafficking of raft protein GPI-APs. CLPTM1L-mediated membrane raft remodeling promotes EGFR activity on the raft domains and drives downstream mTOR/ERK proliferative signaling for tumor progression. Data represent mean ± SD. Two-tailed Student’s *t*-test in (c and f). Log-rank test in (g). **P < 0.01; ***P < 0.001.

To investigate the *in vivo* function of CLPTM1L in cancer, we established a doxycycline-inducible knockdown system in GBM cells and assessed tumor growth in mice after orthotopic implantation of these engineered cells (Fig. 6a–c). In the cells, doxycycline treatment specifically decreased *CLPTM1L* mRNA level and significantly reduced cell viability in GBM cells harboring the inducible sh*CLPTM1L* plasmid (Supplementary Fig. S4g and h). Consistently, in mouse models, compared to the non-targeting control, doxycycline-diet administration significantly reduced the CLPTM1L protein in xenograft tumors expressing knockdown shRNAs (Fig. 6d), resulting in a marked impairment of tumor growth, with only approximately 20% of tumor-positive area detected (Fig. 6b and c). Western blot analysis further confirmed that the intensity of pEGFR-Y1068, pAKT-S473, and pS6-S235/236 was remarkably reduced in *CLPTM1L-*depleted tumor samples (Fig. 6d). Importantly, consistent with the loss of total EGFR from the plasma membrane of cultured cells (Fig. 4k–n), pEGFR-Y1068 signal on the cell surface in tumors was dramatically attenuated after *CLPTM1L* depletion (Fig. 6e and f**)**, further supporting the inhibition of EGFR-mTORC1/2 signaling *in vivo*. Consistent with these results in EGFR-mediated proliferative signaling and tumor growth, mice bearing tumors with *CLPTM1L* depletion exhibited significantly extended survival (Fig. 6g). These *in vivo* results further demonstrate that CLPTM1L promoted oncogenic RTK signaling and drove GBM tumor progression. Together, our data suggest that CLPTM1L, as an ER-localized lipid scramblase, regulates cellular lipid homeostasis and modulates membrane raft lipids and GPI-APs, thereby driving cell proliferation and tumor progression by activating EGFR and downstream signaling (Fig. 6h).

## Discussion

The plasma membrane is the primary organizer of receptor-mediated proliferative signaling in cancer cells, where growth factor receptors assemble within lipid rafts to form functional signaling platforms [6, 7, 16]. In this study, we uncover a specific role of CLPTM1L in remodeling the cell membrane raft domains and driving proliferative signaling in cancer. Depletion of *CLPTM1L* markedly reduced key raft components, including glycosphingolipids, PS, and GPI-APs, which are required for EGFR activation and cancer cell proliferation. Importantly, CLPTM1L alone is sufficient to enhance cell proliferation and EGFR signaling in non-cancer cells, suggesting a high dependency of proliferating cells on CLPTM1L-mediated metabolic and signaling axis. Although CLPTM1L has been linked to chemotherapeutic drug resistance and tumor progression in multiple cancers [30–33], its specific molecular functions have remained controversial. Our results establish critical function of CLPTM1L in membrane raft formation and oncogenic EGFR signaling in GBM. As lipid rafts play important roles in diverse cellular processes, our findings may provide valuable insights into the function of CLPTM1L in cancer pathogenesis. The frequent amplification of CLPTM1L across diverse cancers, along with its potential regulation of many RTKs localized in raft domains [6, 10, 34], underscores CLPTM1L as a potential therapeutic target for a broad spectrum of malignancies. Of note, a potential ligand-binding pocket in the predicted CLPTM1L structure raises the possibility of developing a specific small-molecule inhibitor for targeted cancer therapy.

Our work demonstrates a coordinated regulation mechanism of membrane raft lipids and proteins, which is critical for the activation of EGFR signaling in cancer. The formation of functional lipid raft domains relies on complex lipid−lipid and lipid−protein interactions between their components [2, 7, 8]. ER is the primary site of raft protein and lipid biosynthesis, which can be then transported to the Golgi complex for further modification. GPI-APs and glycosphingolipids appear to share similar trafficking and modification pathways, potentially influencing their membrane localization and abundance [28, 35]. Hex3Ceramide represents the membrane lipid with the most pronounced decrease upon *CLPTM1L* depletion, consistent with known metabolic interplay between GPI-APs and glycosphingolipids [28]. This connection is further supported by evidence that sphingolipid metabolism regulates GPI-AP sorting from the ER in yeast [36], suggesting a conserved mechanism. Therefore, CLPTM1L may coordinately regulate sphingolipid metabolism and GPI-AP biosynthesis, although the detailed mechanisms remain to be elucidated. Within lipid rafts, ganglioside GM1 and GPI-APs not only colocalize but also engage in physical interactions [37]. Moreover, our observation of reduced intensity of cell membrane PS aligns with the transbilayer coupling between inner leaflet PS and GPI-AP rafts [38]. Collectively, CLPTM1L-mediated regulation of membrane raft components, including glycosphingolipids, PS, and GPI-APs, may form an essential interaction network for lipid raft formation, thereby driving EGFR activation and downstream signaling in cancer cells. This mechanism is consistent with our and others’ previous findings of raft lipid-mediated RTK activation [12, 17], although the interactions and dependencies among these lipids, such as saturated phospholipids and glycosphingolipids, require further investigation. Membrane raft domains are important across cell types, including immune cells [39]. Future research should explore the impact of CLPTM1L-mediated membrane remodeling within an immune-competent microenvironment.

CLPTM1L plays a critical role in modulating cellular lipid homeostasis in cancer cells. This may be primarily through its function as an ER lipid scramblase, which facilitates the translocation of diverse phospholipids and GPI precursors [40]. The decrease of Hex3Cer upon *CLPTM1L* depletion may be attributed to the shared metabolic and trafficking machineries between GPI-APs and glycosphingolipids [35]. This aligns with the coordinated increase in Hex1Cer, a substrate for Hex3Cer biosynthesis, highlighting the involvement of CLPTM1L in glycosphingolipid biosynthesis or trafficking from the Golgi to the plasma membrane. Moreover, the altered profiles of PUFA-containing phospholipids and CEs in cancer cells suggest that CLPTM1L is essential for maintaining phospholipid asymmetry across the ER membrane, a process crucial for lipid homeostasis. These findings are consistent with lipid phenotypes observed in other ER-localized scramblases [41, 42], such as the transmembrane protein 41B (TMEM41B), although the specific phospholipid substrates of CLPTM1L in cancer may require further characterization. As an increase in CEs was observed, cholesterol metabolism may also contribute to cell membrane remodeling of *CLPTM1L*-depleted cancer cells. Importantly, the ability of CLPTM1L to limit lipid storage in droplets may further enhance cell proliferation by ensuring efficient utilization of limited substrates for rapid tumor growth [23]. Notably, the high expression of *CLPTM1L* in the human liver warrants further investigation into its potential role in metabolic homeostasis under physiological conditions.

In summary, we reveal an ER-localized regulator that modulates plasma membrane lipid domain formation, a process essential for establishing the sustaining proliferative signaling in cancer cells. This dependency on membrane lipid metabolism and signaling presents a promising therapeutic target for diverse cancers. In addition, our findings may contribute to a deeper understanding of plasma membrane architecture dynamics, with possible implications for a range of biological contexts, including signal transduction, cell proliferation, and tissue metabolic homeostasis in both normal and pathological states.

### Limitations of the study

Although our data support the regulation and a functional role of HexCer-associated raft remodeling in CLPTM1L-mediated signaling, we have not established a direct biochemical mechanism by which CLPTM1L controls HexCer biosynthesis. Broader lipid alterations in the ER, including increased CEs and lipid droplets, may contribute to the membrane raft remodeling. The *in vivo* mouse experiments were performed in an orthotopic xenograft model rather than an immunocompetent model, which does not capture the role of CLPTM1L in tumor−immune interactions. Finally, the proposed druggability of CLPTM1L remains preliminary and requires future experimental validation. Addressing these limitations will be critical for advancing our understanding of CLPTM1L-mediated membrane remodeling and its therapeutic potential.

## Materials and methods

### Cell lines

Human cell lines A172, LN229, SVG-P12, PC-9, H1437, HeLa, and HEK-293T were from the Cell Bank of Chinese Academy of Sciences, and RPE1, U87, and T98G were obtained from the American Type Culture Collection. All adherent cells were maintained in DMEM supplemented with 10% FBS (VivaCell) and 1% penicillin/streptomycin (Gibco). GBM neurosphere lines were cultured in DMEM/F12 medium supplemented with 1× B27 (Gibco), 1× Glutamax (Gibco), 1 µg/mL heparin (Sigma), 20 ng/mL EGF (Sigma), and 20 ng/mL FGF (Peprotech) as previously described [43]. The A172EGFRvIII and U87EGFRvIII isogenic cell lines were established by stably expressing EGFRvIII in commercial cell lines [17]. All cell lines were confirmed to be mycoplasma-negative and maintained at 37°C in a humidified incubator with 5% CO_2_.

### Mouse models

For orthotopic mouse models, GBM cells were engineered to carry inducible shRNAs and intracranially injected as previously described [17]. Briefly, A total of 2.0 × 10^5^ cells in 5 µL PBS were intracranially injected into 5-week-old female athymic nude mice (GemPharmatech). Doxycycline-containing diet (HY-14889, Research Diets) was given from Day 4 after injection. Mouse weight was monitored once per week, and survival was recorded until the onset of neurologic symptoms. Five mice per group were sacrificed for tumor sections and hematoxylin and eosin (H&E) staining, followed by tumor size quantification, and nine mice per group were injected for the survival analysis. All experiment protocols were approved by the Institutional Animal Use and Care Committee at Fudan University.

### Plasmids and lentivirus infections

Lentiviral shRNA plasmids were cloned with two individual targeting sequences using a pLKO.1 vector (Supplementary Table S3). Lentivirus expression plasmids for *CPTLM1L* and *A4GALT* were generated by cloning the coding sequence into a pLVX-Puro vector. Coding sequence for 3×HA was inserted in-frame with *CLPTM1L* to generate the N-terminal tagged *CLPTM1L* expression plasmid. EGFRvIII and AKT1-E17K were constructed as previously described [17, 18]. For GPI-mScarlet, the mScarlet coding sequence was fused in-frame downstream of the CD59 N-terminal signal peptide and upstream of the CD59 C-terminal GPI-anchoring signal. The resulting fusion protein was further inserted into a pLV-BSD backbone. Cells were infected with lentivirus and selected with puromycin or blasticidin before further experiments.

### Cell viability, cell count, and colony formation assay

Cell viability was measured using a Cell Counting Kit-8 assay kit (HY-K0301, MedChemExpress), and absorbance at 450 nm was recorded by a Tecan Spark Plate Reader. Cell numbers were counted using an automatic cell counter. Crystal violet-stained colonies were imaged using a ChemiDoc MP imaging system (Bio-Rad) and counted with ImageJ software. At least three biological replicates were performed.

### RNA extraction and qRT-PCR analysis

Total RNA was extracted using a RNeasy Mini kit (Vazyme), and reverse transcription was performed with a cDNA synthesis kit (TOYOBO). Samples mixed with cDNA, primers, and SYBR Green Supermix (Yeasen) were amplified on a CFX96 real-time PCR detection system (Bio-Rad). The data were analyzed by the ΔΔC_t_ method and further normalized to the reference gene *TBP* and the indicated control group. All sequences of primers are listed in Supplementary Table S3.

### Immunofluorescence staining

Cells were seeded on coverslips for the indicated treatments. After washing with PBS, cells were fixed with 4% paraformaldehyde and permeabilized with 0.2% Triton X-100. The cells were then blocked with 2% BSA and incubated with the primary antibodies overnight at 4°C (anti-EGFR, 4267, Cell Signaling, 1:200; anti-HA, 3724, Cell Signaling, 1:1000). After washing with PBS for three times, the cells were further incubated with the fluorescent antibodies (A11029, A11034, and A11037, Invitrogen, 1:1000) at room temperature for 1 h. For cell surface GM1 staining, live cells were pre-chilled on ice for 15 min and incubated with 2 μg/mL CTxB-488 conjugates (C34775, Thermo Fisher) on ice for 30 min. The cells were washed with cold PBS and processed for further antibody staining. Lipid droplets were stained with BODIPY 493/503 (D3922, Thermo Fisher). Samples were mounted with an antifade reagent containing 4’,6-diamidino-2-phenylindole (DAPI) and imaged on a Zeiss LSM 880 confocal microscope.

### Lipidomics analysis

T98G cells were infected with shRNA lentivirus and selected with puromycin for 5 days. Cell pellets were harvested, and lipids were extracted using the Matyash method. Briefly, cell pellets were resuspended in 200 µL of HPLC-grade water and transferred into glass tubes. After vortexing with 1.5 mL methanol for 1 min, 5 mL methyl tert-butyl ether (MTBE) was further added and vortexed for another minute. After 1 h of shaking on a horizontal shaker for lipid extraction, 1.25 mL of HPLC-grade water was added to achieve phase separation by centrifugation. The top organic phase containing lipids was transferred into a clean tube and dried under nitrogen gas. Samples were frozen at −80°C until the following analysis. LC/MS analysis was performed on a Vanquish Flex HPLC system coupled to a Thermo Orbitrap Exploris 480 mass spectrometer. Cell numbers of each sample were counted and applied for final data normalization. Five biological replicates were prepared for both control and *CLPTM1L* knockdown cells. The lipidomics data are listed in Supplementary Table S1.

### Antibodies, western blot, and immunohistochemistry analysis

Cells or homogenized tumor samples were lysed on ice for 30 min with RIPA buffer supplemented with protease and phosphatase inhibitors. The supernatants were mixed with SDS sample buffer and incubated on ice for another 30 min before western blot ana­lysis. Membranes were blocked with 5% BSA and then incubated with the following primary and secondary antibodies: CLPTM1L (HPA014791, Sigma), EGFR (4267, Cell Signaling), pEGFR-Y1068 (3777, Cell Signaling), pEGFR-Y1173 (4407, Cell Signaling), pAKT-S473 (4060, Cell Signaling), pS6-S235/236 (4858, Cell Signaling), pERK1/2 (4370, Cell Signaling), CD59 (ab9183, Abcam), β-actin (3700, Cell Signaling), anti-rabbit IgG HRP-linked antibody (7074, Cell Signaling), and anti-mouse IgG HRP-linked antibody (7076, Cell Signaling). The blots were then imaged on a ChemiDoc imaging system (Bio-Rad). For chloroquine treatments, cells infected with the indicated shRNAs were selected with puromycin for 3 days and then incubated for an additional 6 h in the absence or presence of 50 μmol/L chloroquine (MCE, HY-17589A) before collection. For immunohistochemistry (IHC) analysis, brain sections were incubated with pEGFR-Y1068 antibody (3777, Cell Signaling) overnight, and scanned images were quantified using ImageJ.

### Patient dataset analysis

Annotated and processed datasets from TCGA and CGGA for mRNA expression, gene copy numbers, and patient survival were downloaded from the GlioVis Data Portal, GEPIA, or the cBioPortal [26, 44, 45]. The Rembrandt glioma dataset was applied in comparing differential *CLPTM1L* expression in nontumor, LGG, and GBM samples. Overall survival of patients with high or low *CLPTM1L* expression was statistically compared using the TCGA and CGGA GBM datasets. *P-*values and patient numbers of each cohort were indicated. The information regarding patient IDs and *CLPTM1L* mRNA levels for the *CLPTM1L*-high and *CLPTM1L*-low cohorts of TCGA glioma patients is listed in Supplementary Table S2.

### Statistical analysis

Statistical analysis was performed using the GraphPad Prism 10 software unless specified in the method. Specifically, the log-rank (Mantel-Cox) test was used to evaluate the survival effects in patients and mice. The unpaired two-tailed Student’s *t*-test was performed to assess differences between two experimental groups, and an analysis of variance (ANOVA) followed by appropriate multiple comparison tests was applied to compare three or more groups. *P-*values <0.05 are defined to be significant. Data are presented as mean ± SD or SEM, and detailed information, including numbers of samples or biological replicates, applied statistical tests, and *P-*values, is indicated in each figure legend.

## Supplementary Material

loag012_Supplementary_Data

## Data Availability

All data supporting the findings of this study are available within the supplementary material and from the corresponding author upon reasonable request.
