# Supplementary material for: CLPTM1L modulates membrane lipid rafts to promote tumor EGFR signaling
Source: Life Metab. 2026 May 20;5(4):loag012. doi: 10.1093/lifemeta/loag012 (PMC13275298; doi:10.1093/lifemeta/loag012)
Supplement: loag012_Supplementary_Data [file loag012_supplementary_data.zip › Supplementary_Figures_and_Tables_revised - tu.pdf]

Supplementary Figures and Tables

Figure S1.

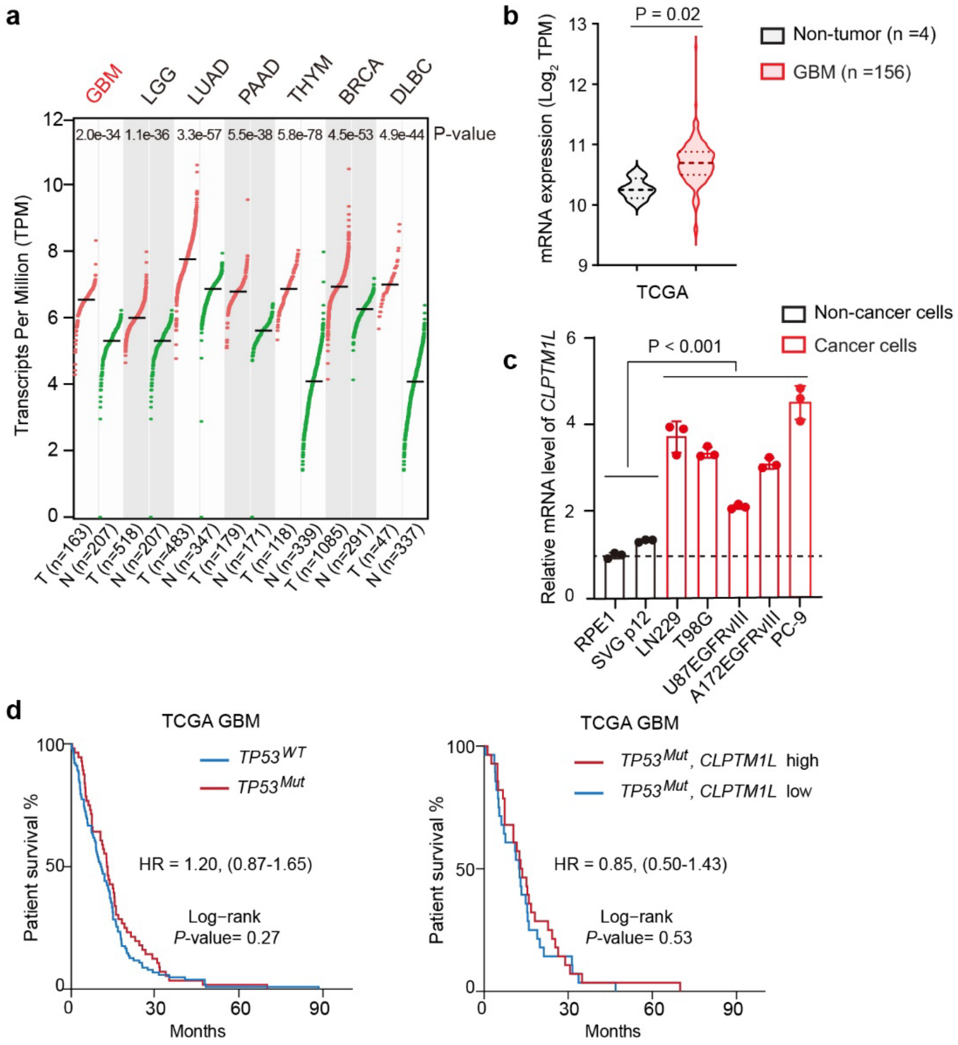

Supplementary Figure S1 *CLPTM1L* is highly expressed in cancer.

(a) The mRNA levels of *CLPTM1L* in TCGA tumor samples and matched GTEx normal samples. (b) The mRNA levels of *CLPTM1L* in non-tumor samples and GBM tumor samples from the TCGA GBM dataset. (c) The mRNA levels of *CLPTM1L* in multiple non-cancer and cancer cell lines. (d) Overall survival of patients with *TP53* alterations or with high or low *CLPTM1L* expression in the TCGA GBM cohort. Data represent mean ± SD in (c). Two-tailed Student's *t*-test in (a and b). ANOVA followed by Tukey's multiple comparisons test in (c). Log-rank test in (d).

Figure S2.

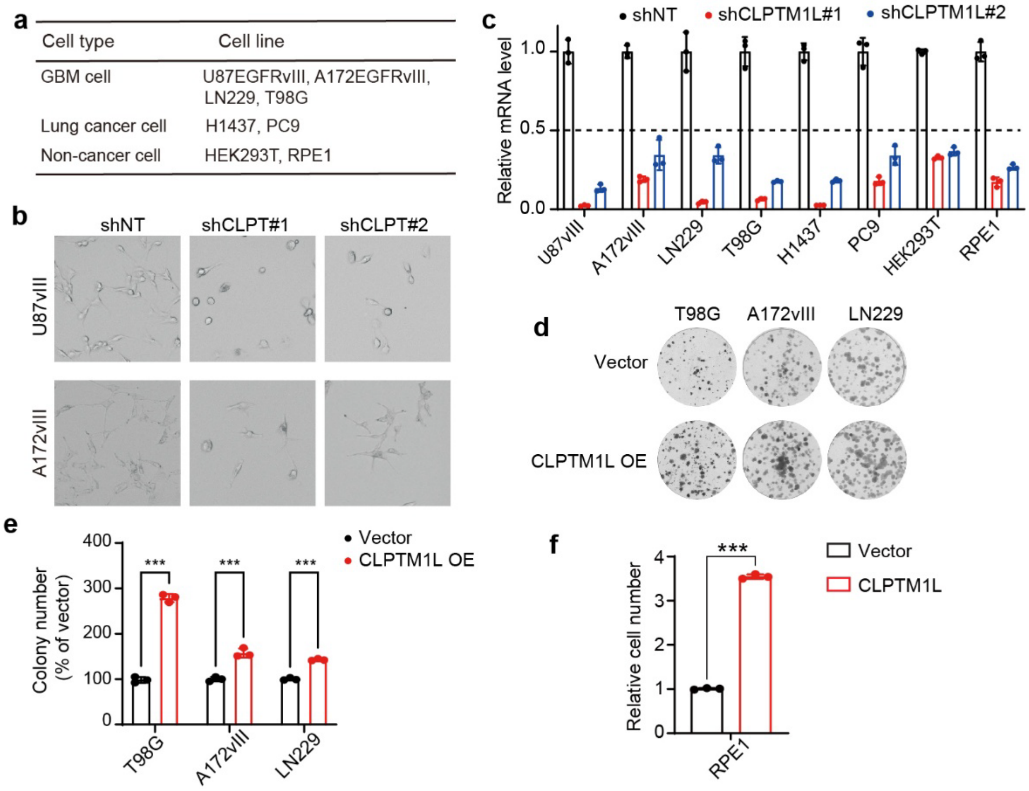

Supplementary Figure S2 CLPTM1L promotes cell proliferation of cancer cells and non-cancer cells.

(a) Cell lines applied in the functional study. (b) Representative images of cells expressing control or *CLPTM1L* shRNAs. (c) The mRNA level of *CLPTM1L* showing knockdown efficiency in cell lines. (d and e) Colony formation of cancer cells with *CLPTM1L* overexpression. (f) Relative cell numbers of RPE1 cells with *CLPTM1L* overexpression. Data represent mean ± SD. ANOVA followed by Tukey's multiple comparisons test in (c and e). Two-tailed Student's *t*-test in (f). $^{***}P < 0.001$.

Figure S3.

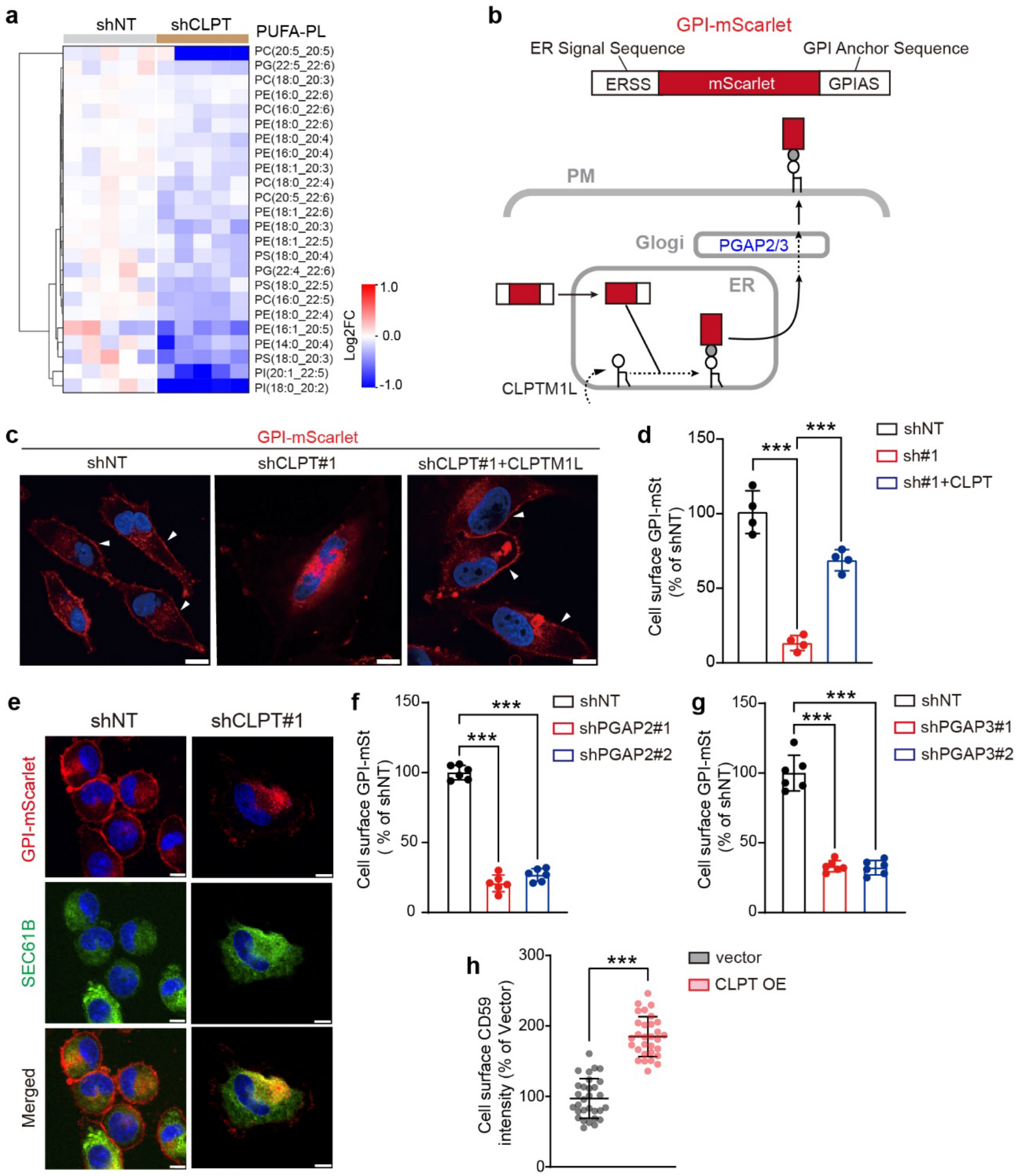

Supplementary Figure S3 CLPTM1L regulates membrane lipids and promotes plasma membrane localization of GPI-APs in GBM cells.

(a) Heatmap of significantly altered PUFA-containing phospholipid species in T98G cells. (b) Schematic diagram of GPI-mScarlet marker and the biosynthesis and membrane trafficking of GPI-APs. (c) GPI-mScarlet merged with DAPI in T98G cells with *CLPTM1L* knockdown and genetic rescue. Scale bar, 5 μm. (d) Quantification of the percentage of cell membrane GPI-mScarlet positive cells in (c). (e) Staining of GPI-mScarlet with ER marker Sec61B and DAPI in T98G cells. Scale bar, 5 μm. (f and g) Percentage of cell surface GPI-mScarlet positive cells with *PGAP2* or *PGAP3* knockdown. (h) Relative intensity of cell surface CD59 in T98G cells with vector or *CLPTM1L* overexpression. Data represent mean ± SD. ANOVA followed by Tukey's multiple comparisons test in (d, f-h). Two-tailed Student's *t*-test in (h). ***$P < 0.001$.

Figure S4.

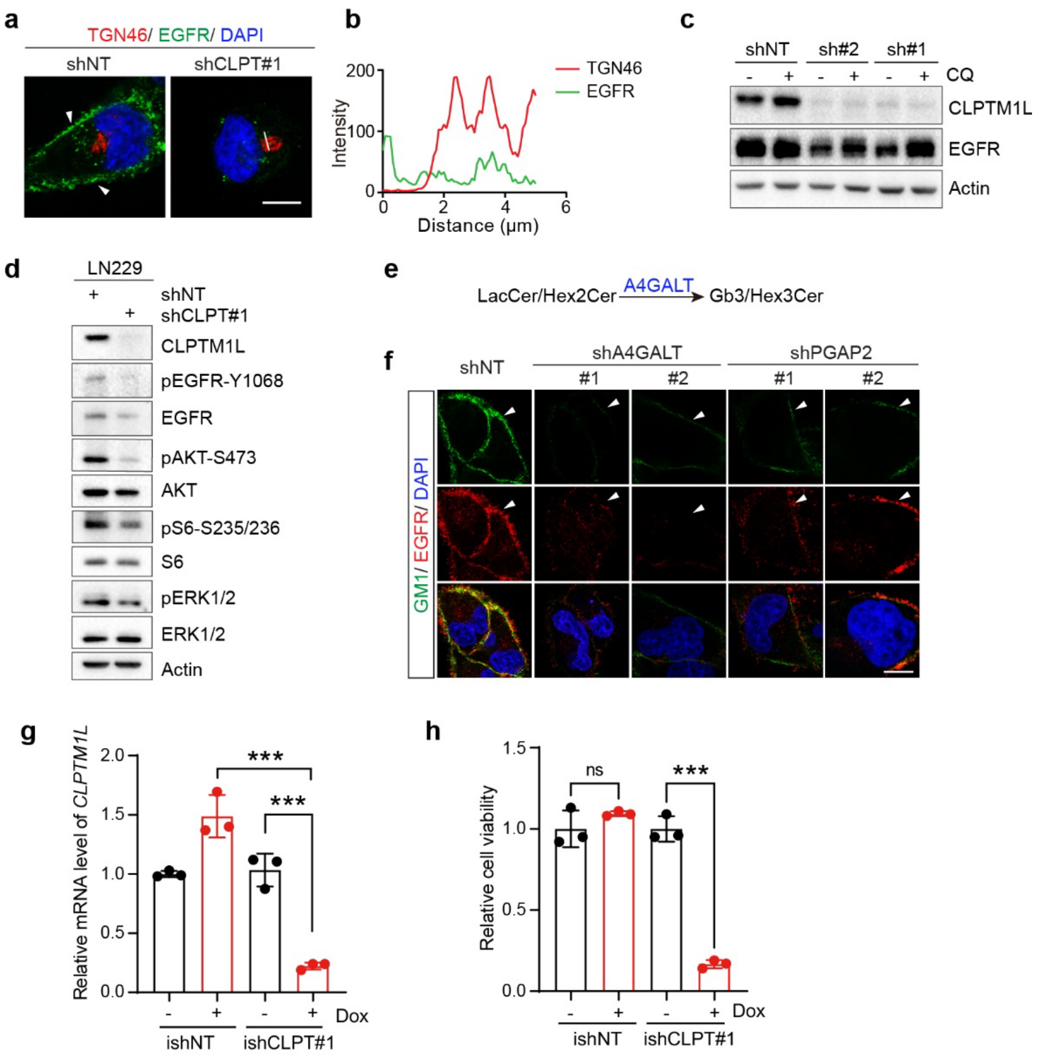

Supplementary Figure S4 CLPTM1L promotes EGFR signaling and cell proliferation in GBM cells.

(a) Staining of EGFR and the Golgi marker TGN46 in T98G cells. Scale bar, 10 μm. (b) Intensity quantification of ER and TGN46 along the indicated lines in (a). (c) Western blot analysis of EGFR protein in the control and *CLPTM1L* knockdown cells with or without chloroquine (CQ) treatment. (d) Western blot analysis of EGFR protein in LN229 cells with *CLPTM1L* knockdown. (e) A4GALT in Hex3Cer biosynthesis. (f) Staining of GM1 and cell surface EGFR in T98G cells. Scale bar, 10 μm. (g) Relative mRNA level of *CLPTM1L* in GBM cells with or without doxycycline inducible. (h) Relative cell viability of GBM cells with or without doxycycline inducible. DAPI staining indicates cell nuclei. Data represent mean ± SD. ANOVA followed by Tukey's multiple comparisons test in (g andh). ns, not significant; $^{***}P < 0.001$.

Supplementary Table S1 The lipidomics data of GBM cells with non-targeting control or *CLPTM1L* knockdown.

Supplementary Table S2 The information on the *CLPTM1L*-high and *CLPTM1L*-low patient cohorts from TCGA glioma dataset.

Supplementary Table S3 The sequence of shRNAs and qRT-PCR primers.

Name	Sequence
CLPTM1L#1 shRNA	CCAGCCAAGTGCAACTTGAAT
CLPTM1L#2 shRNA	CAGTTTCTGGAAGAAGAAGAA
PGAP2#1 shRNA	CCATCCAGTTTCTGGCCTTTA
PGAP2#2 shRNA	CGGCTTCTTCTTCTGCATCAT
PGAP3#1 shRNA	AGAATCGCCGTGAACCTTGGC
PGAP3#2 shRNA	GGAATCAGAGGACAAGTTCAA
A4GALT#1 shRNA	CACGGACTTCATTGTTCTCAA
A4GALT#2 shRNA	AGGAGGCTGTTGGTAGGAAAG
CLPTM1L-fwd	TCCTTCACCAGCTTGGTGGTGG
CLPTM1L-rev	CTTCCACATTCAAGACCAGGTCG
TBP-fwd	CCACTCACAGACTCTCACAAC
TBP-rev	CTGCGGTACAATCCCAGAACT
